# Gene therapy for alpha 1-antitrypsin deficiency with an oxidant-resistant human alpha 1-antitrypsin

**DOI:** 10.1172/jci.insight.135951

**Published:** 2020-08-06

**Authors:** Meredith L. Sosulski, Katie M. Stiles, Esther Z. Frenk, Fiona M. Hart, Yuki Matsumura, Bishnu P. De, Stephen M. Kaminsky, Ronald G. Crystal

**Affiliations:** Department of Genetic Medicine, Weill Cornell Medical College, New York, New York, USA.

**Keywords:** Pulmonology, Therapeutics, Gene therapy, Genetic diseases

## Abstract

Alpha 1-antitrypsin (AAT) deficiency, a hereditary disorder characterized by low serum levels of functional AAT, is associated with early development of panacinar emphysema. AAT inhibits serine proteases, including neutrophil elastase, protecting the lung from proteolytic destruction. Cigarette smoke, pollution, and inflammatory cell–mediated oxidation of methionine (M) 351 and 358 inactivates AAT, limiting lung protection. In vitro studies using amino acid substitutions demonstrated that replacing M351 with valine (V) and M358 with leucine (L) on a normal M1 alanine (A) 213 background provided maximum antiprotease protection despite oxidant stress. We hypothesized that a onetime administration of a serotype 8 adeno-associated virus (AAV8) gene transfer vector coding for the oxidation-resistant variant AAT (A213/V351/L358; 8/AVL) would maintain antiprotease activity under oxidant stress compared with normal AAT (A213/M351/M358; 8/AMM). 8/AVL was administered via intravenous (IV) and intrapleural (IPL) routes to C57BL/6 mice. High, dose-dependent AAT levels were found in the serum and lung epithelial lining fluid (ELF) of mice administered 8/AVL or 8/AMM by IV or IPL. 8/AVL serum and ELF retained serine protease–inhibitory activity despite oxidant stress while 8/AMM function was abolished. 8/AVL represents a second-generation gene therapy for AAT deficiency providing effective antiprotease protection even with oxidant stress.

## Abstract 2

A gene transfer-based therapeutic to deliver oxidant-resistant alpha 1-antitrypsin (AAT) protects mice with AAT deficiency from lung destruction.

## Graphical abstract

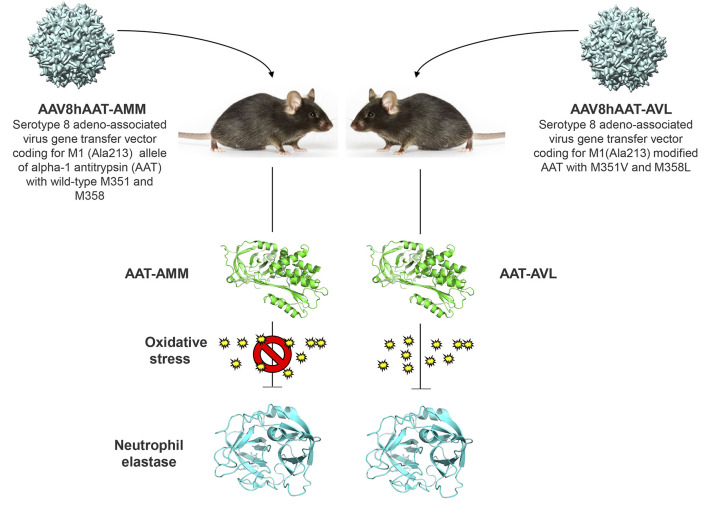

## Introduction

Alpha 1-antitrypsin (AAT) deficiency is a common autosomal recessive disorder affecting 1/2000 to 1/5000 individuals, about 90,000 people in the United States (1–5). The primary manifestation of AAT deficiency is early-onset panacinar emphysema. The disease, which is accelerated by smoking, presents at ages 35 to 40 in smokers compared with ages 55 to 60 in nonsmokers (1, 3, 6–8). AAT is a serine protease inhibitor that functions to inhibit neutrophil elastase (NE) as well as other neutrophil-released serine proteases, including proteinase 3, α-defensins, and cathepsin G (1, 2, 6, 9–13). AAT is produced predominantly in the liver and diffuses across the lung from the circulation (1, 2, 4, 6, 9, 14–16). In the lung, AAT is also produced locally by monocytes, macrophages, alveolar epithelial type 2 cells, and bronchial epithelial cells (17–19). However, hepatocytes produce the vast majority of functional AAT as AAT-deficient patients who receive liver transplants convert to the donor AAT phenotype (20–22). Low serum levels of AAT, and thus in the alveolar structures and alveolar epithelial lining fluid (ELF), are associated with an imbalance between AAT and neutrophil-released proteases, leading to the slow destruction of the lung parenchyma (1, 2, 4, 6, 9, 14, 16, 23).

AAT deficiency is caused by mutation in the *SERPINA1* gene, a member of the serpin family of antiproteases (3, 5, 24, 25). The normal M alleles are present in more than 98% of the population (3, 5). Although there are more than 120 known alleles of AAT, individuals homozygous for the Z allele (E342K) account for more than 95% of clinically recognized cases of AAT deficiency (2, 4, 6, 23, 26–28). The Z allele causes the AAT protein to polymerize in hepatocytes, limiting secretion into the circulation, resulting in plasma levels 10% to 15% of the levels of normal M homozygotes (4, 5, 7, 29–31).

AAT binds serine proteases through its active site centered at methionine (M) 351 and 358 (M358, M351) through an irreversible interaction that inactivates both the protease and AAT (32, 33). These M residues in AAT are inactivated by exogenous oxidants, including cigarette smoke and air pollutants and endogenous oxidants from activated inflammatory cells (34, 35). Under oxidizing conditions, the M351 and M358 in the active site are oxidized to methionine sulfoxide, significantly reducing the ability of AAT to function (36–42). AAT in lung ELF of smokers has markedly reduced ability to inhibit NE compared with nonsmokers (34, 43, 44); this sensitivity of AAT to oxidants is the reason behind the early occurrence of emphysema in smokers with AAT deficiency (44, 45).

The current therapy for AAT deficiency is augmentation with weekly infusions of purified AAT from pooled human plasma (9, 46–48). The infusion of AAT protein normalizes AAT levels in the serum and corrects the deficiency of levels in lung ELF, protecting the alveoli from proteolytic destruction (9, 46, 47). While AAT augmentation therapy is safe and reduces the rate of lung destruction in AAT-deficient individuals (49–52), protein augmentation therapy requires a treatment regimen of weekly parenteral infusions and has the theoretical risks associated with human plasma-derived products, such as allergic reactions and viral contamination (48, 53, 54).

Gene therapy is a strategy to circumvent both the requirement of weekly administration of the AAT protein and the sensitivity of AAT to oxidants. First-generation gene therapy strategies for the treatment of AAT by our group and others have used the coding sequence from the normal M1 allele, which is an effective inhibitor of serine proteases but has M351 and M358 residues that are vulnerable to oxidation (55–73). Our lab and others have demonstrated that changing either the active site M351 or M358 to valine (V) or leucine (L) can prevent oxidation while maintaining serine protease–inhibitory capacity (40, 74–81). Based on these studies, we hypothesized that a second-generation adeno-associated virus serotype 8 (AAV8) gene transfer vector coding for an oxidant-resistant AAT would maintain function even under oxidizing conditions. The data demonstrate that in vivo gene therapy with AAT with modified M351 to V and M358 to L (AAT-AVL) results in persistent expression of AAT at high levels in serum and lung ELF in mice. The vector-produced AAT-AVL retains function in the presence of excess oxidants and represents a second-generation strategy to treat AAT deficiency.

## Results

### Optimization of an oxidant-resistant AAT gene.

**Figure 1:**
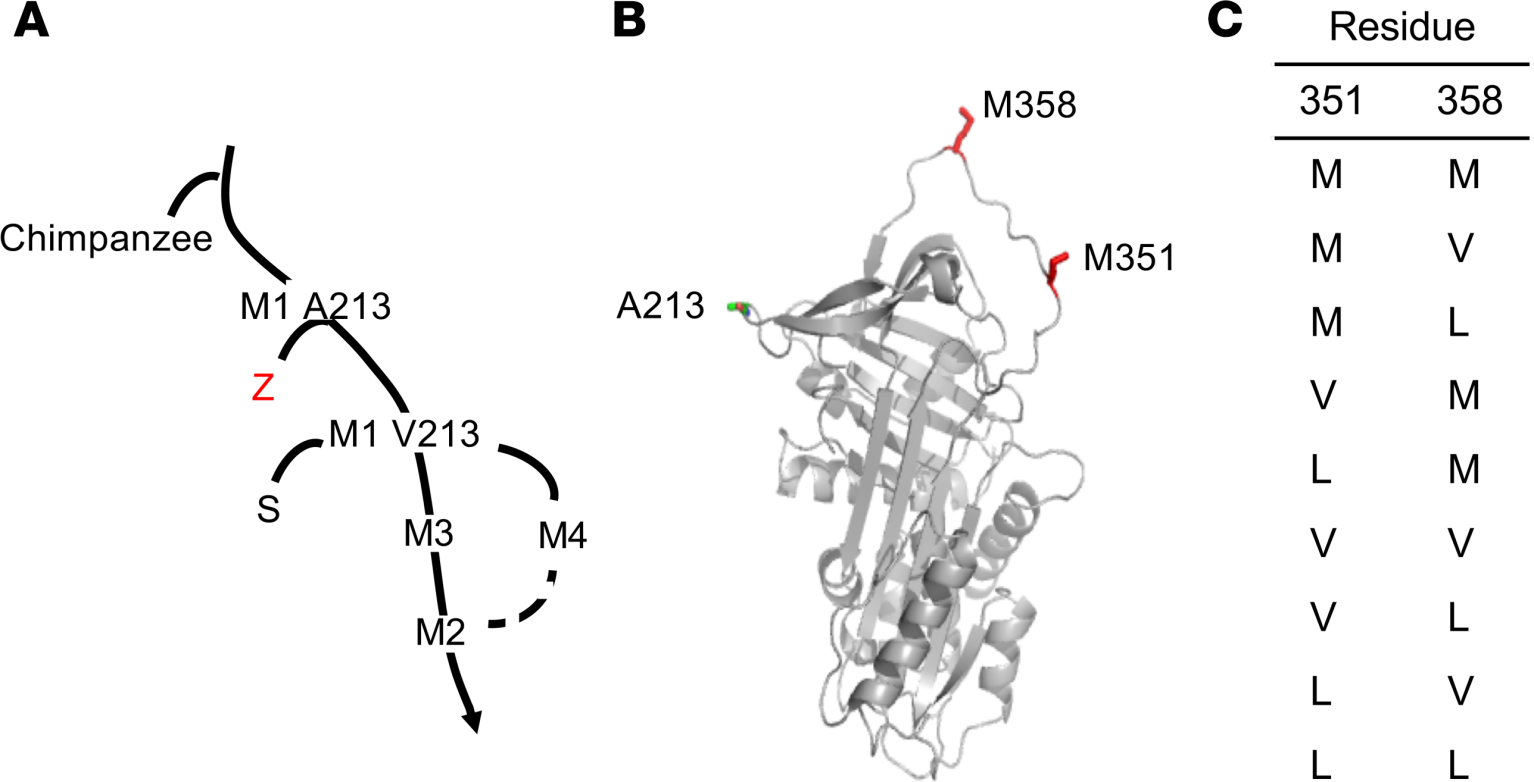
Design of a second-generation gene therapy for AAT deficiency. (**A**) Phylogenetic tree of AAT evolution (101, 102). M1(A213), M1(V213), M2, M3, and M4 are all normal variants. The common Z variant is derived from M1(A213). The less common S deficiency allele is derived from M1(V213). Since more than 95% of all AAT-deficient individuals are ZZ homozygotes, the M1(A213) normal variant was used as the base for the second-generation AAT gene therapy constructs to minimize immune responses to the therapy. (**B**) Three-dimensional schematic of human AAT with the A213 normal variant and active site methionine residues (M351, M358) indicated (red). (**C**) AAT variants tested.

Individuals homozygous for the Z allele account for more than 95% of clinically recognized cases of AAT deficiency (1, 4–6, 23, 27, 28). Because the Z allele is derived from the normal M1 alanine 213 (A213) allele (Figure 1A), we used the normal AAT M1(A213) coding sequence as the base of the transgene expression cassette, thus minimizing the theoretical possibility of immunity generated against the AAT transgene. Prior studies have shown that substitution of V or L for M at positions 351 or 358 in the AAT active site can prevent inactivation of AAT by oxidation (40, 74–78, 80–82) (Figure 1B). To determine the best combination of V and L substitutions for M351 and M358, all combinations of single and double substitutions were made by site-directed mutagenesis (Figure 1C). All AAT variants were produced in vitro and tested for their ability to inhibit NE and cathepsin G in the absence or presence of oxidants using NE and cathepsin G inhibition assays.

**Figure 2:**
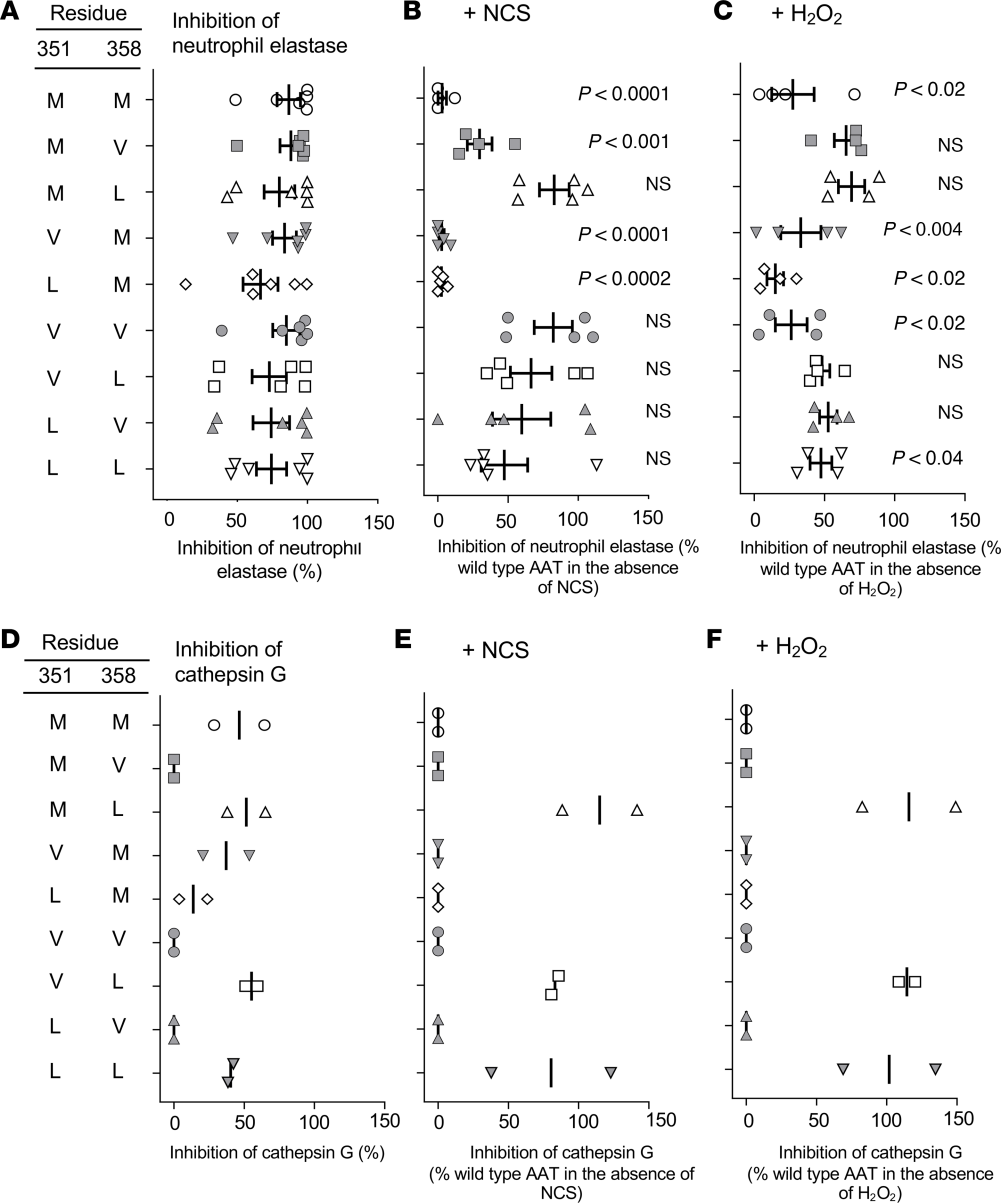
In vitro comparison of the ability of modified variants of human AAT to inhibit NE and cathepsin G under normal and oxidizing conditions. All data are presented as the percentage of WT inhibition in the absence of oxidizer. (**A**) NE inhibition by AAT variants under normal conditions (*n* = 6). (**B**) NE inhibition after exposure of AAT variants to 1 mM NCS for 20 minutes (*n* = 4). (**C**) NE inhibition after exposure of AAT variants to 250 mM H_2_O_2_ for 40 minutes (*n* = 4). (**D**) Cathepsin G inhibition by AAT variants under normal conditions (*n* = 2). (**E**) Cathepsin G inhibition after exposure of AAT variants to 1 mM NCS for 20 minutes (*n* = 2). (**F**) Cathepsin G inhibition after exposure of AAT variants to 250 mM H_2_O_2_ for 40 minutes (*n* = 2). Comparison for *P* value is between inhibition under normal conditions and inhibition with either NCS or H_2_O_2_ for each AAT variant. Each assay was performed in triplicate and averaged for each independent experiment. Statistical analysis was performed by ANOVA.

All AAT variants were able to inhibit NE under nonoxidizing conditions at levels similar to the WT (M351/M358) AAT (Figure 2A). However, in the presence of the oxidizer *N*-chlorosuccinimide (NCS), inhibitory activity of WT AAT was completely abolished (Figure 2B). Activity was also abolished for other AAT variants with M at position 358 (V351/M358, L351/M358). In contrast, variants with either V or L substituted in position 358 retained NE-inhibitory activity that was not significantly different from the inhibitory capacity in the absence of oxidant stress. Similar results were also found for NE inhibition in the presence of hydrogen peroxide (H_2_O_2_; Figure 2C). In the absence of oxidants, WT AAT inhibited approximately 40% of the cathepsin G activity (Figure 2D). The variants with V at position 358 (M351/V358, V351/V358, L351/V358) were unable to inhibit cathepsin G while the variants with M or L at position 358 retained this ability. In the presence of NCS, the ability of WT AAT to inhibit cathepsin G was eliminated (Figure 2E). Interestingly, only 3 variants, M351/L358, V351/L358, and L351/L358, inhibited cathepsin G in the presence of NCS. The same 3 variants were also able to inhibit cathepsin G with H_2_O_2_ (Figure 2F).

**Figure 3:**
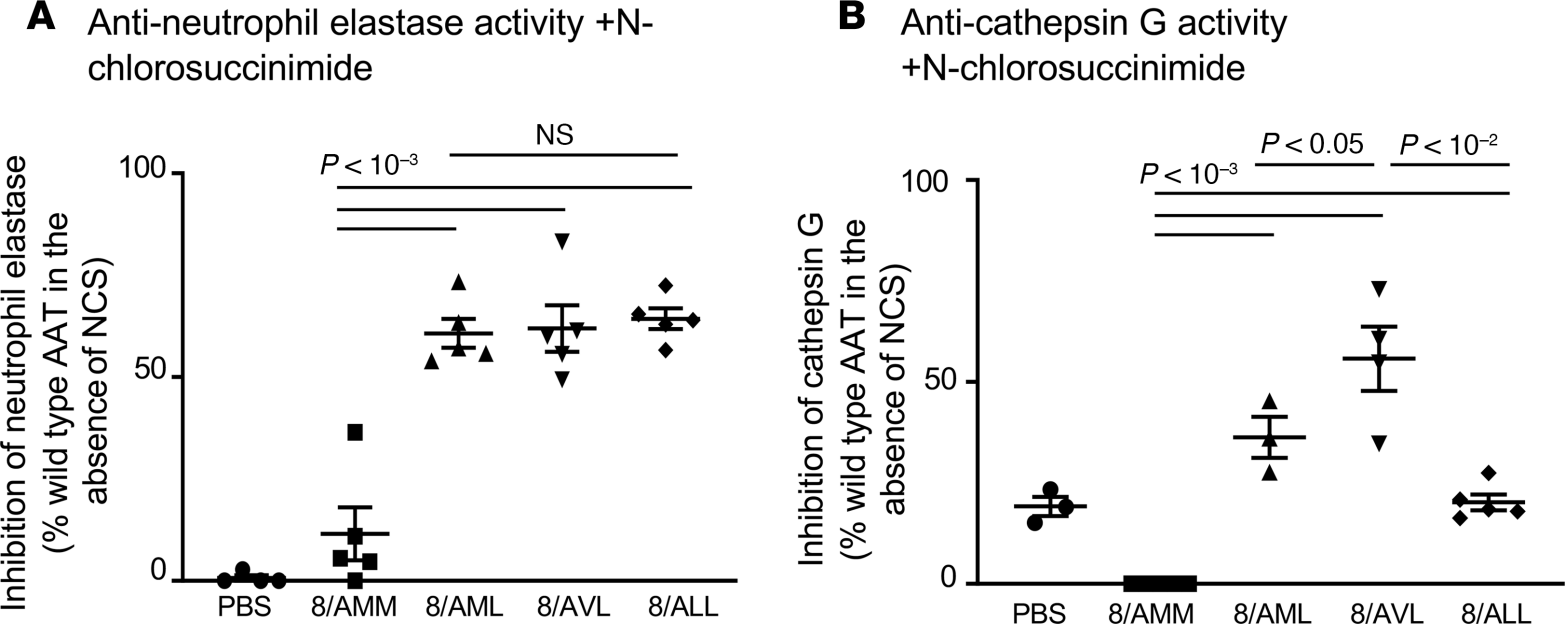
Comparison of in vivo–produced human AAT-modified variants’ ability to inhibit NE and cathepsin G. AAV8 vectors expressing modified AAT variants were administered to C57BL/6 male mice (IV, 10^11^ gc), and serum was collected after 4 weeks. Human AAT levels were quantified by ELISA. An equal amount of each AAT variant was used in the protease inhibition assays. (**A**) Anti-NE activity. AAT variants were exposed to 1 mM NCS for 20 minutes before addition of NE and substrate. NE inhibition is presented as the percentage of WT (8/AMM) inhibition in the absence of oxidizer. (**B**) Anti–cathepsin G activity. AAT variants were exposed to 1 mM NCS for 20 minutes before addition of cathepsin G and substrate. Cathepsin G inhibition is presented as the percentage of WT (8/AMM) inhibition in the absence of oxidizer. Experiments were performed in triplicate and averaged for *n* = 3–5 mice per group, and statistical analysis was performed by ANOVA.

The 3 AAT variants (M351/L358 [8/AML], V351/L358 [8/AVL], and L351/L358 [8/ALL]) that retained NE- and cathepsin G–inhibitory capacity in the presence of NCS and H_2_O_2_ were packaged into AAV8 capsids for testing in mice in vivo (Supplemental Figure 1; supplemental material available online with this article; https://doi.org/10.1172/jci.insight.135951DS1). C57BL/6 mice (*n* = 5 males/group) were administered 10^11^ genome copies (gc) intravenously (IV), and serum was collected after 4 weeks to assess NE- and cathepsin G–inhibitory capacity in the presence of the NCS oxidant. AAT from 8/AMM (A213/M351/M358) could inhibit only 12% of NE activity under oxidizing conditions. In contrast, AAT from 8/AML, 8/AVL, and 8/ALL retained significantly higher NE-inhibitory capacity in the presence of NCS compared with WT 8/AMM (Figure 3A). Cathepsin G–inhibitory activity of WT AAT from 8/AMM was abolished with NCS. In contrast, AAT from 8/AML, 8/AVL, and 8/ALL were all significantly better at inhibiting cathepsin G under oxidizing conditions than 8/AMM (Figure 3B). Overall, the 8/AVL construct performed the best of the 3 variants and was chosen for further studies.

### Binding association rates to NE of AAT-AMM and AAT-AVL.

**Figure 4:**
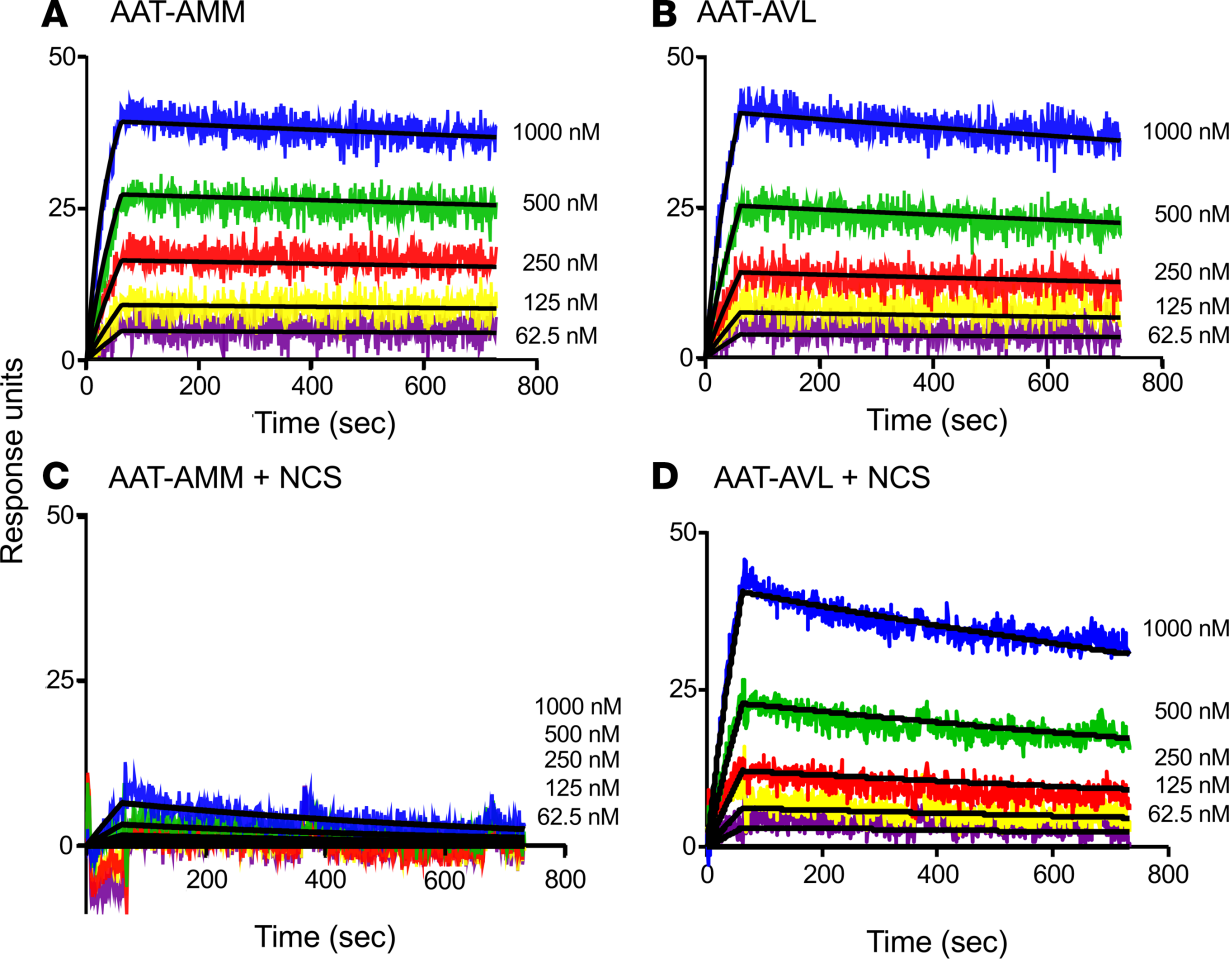
Association rates between human AAT and NE measured by surface plasmon resonance under normal and oxidizing conditions. NE was coupled to the sensor chip, and serial 2-fold dilutions of AAT were flowed across and allowed to bind (AAT: 1000 nM shown in blue, 500 nM shown in green, 250 nM shown in red, 125 nM shown in yellow, 62.5 nM shown in purple). Curve fit to the Langmuir binding model (black line) (98). Binding curves are shown from 1 representative experiment of 3 independent experiments. (**A**) AAT-AMM binding to NE under normal conditions. (**B**) AAT-AVL binding to NE under normal conditions. (**C**) AAT-AMM binding to NE after exposure to 1 mM NCS for 20 minutes. (**D**) AAT-AVL binding to NE after exposure to 1 mM NCS for 20 minutes. See Table 1 for association rates and statistical comparisons.

AAT irreversibly binds and inhibits NE (32, 35). Surface plasmon resonance was used to calculate the association rate constant (*K_a_*) for the interaction between in vitro–produced AAT-AMM or AAT-AVL and NE under normal and oxidizing conditions. Under normal conditions, both AAT-AMM and AAT-AVL bound rapidly to NE with *K_a_* of 3.45 × 10^4^ ± 4.15 × 10^3^ M^–1^s^–1^ for AAT-AMM and 1.60 × 10^4^ ± 5.65 × 10^3^ M^–1^s^–1^ for AAT-AVL (Figure 4, A and B; and Table 1). These association rates are comparable to AAT from normal human serum (*K_a_* = 2.84 × 10^4^ ± 2.17 × 10^3^ M^–1^s^–1^, normal serum vs. AAT-AMM, *P* > 0.7; normal serum vs. AAT-AVL, *P* > 0.2). Strikingly, in the presence of NCS, AAT-AMM showed little binding to NE, while AAT-AVL bound to NE similarly to normal conditions (*K_a_* = 1.59 × 10^4^ ± 5.78 × 10^3^ M^–1^s^–1^; Figure 4, C and D; and Table 1). AAT-AMM NE-inhibitory capacity was abolished under oxidizing conditions because there was no binding to NE, while AAT-AVL NE-binding and -inhibitory capacity was retained.

### Evaluation of a second-generation oxidant-resistant AAT gene therapy in vivo.

**Figure 5:**
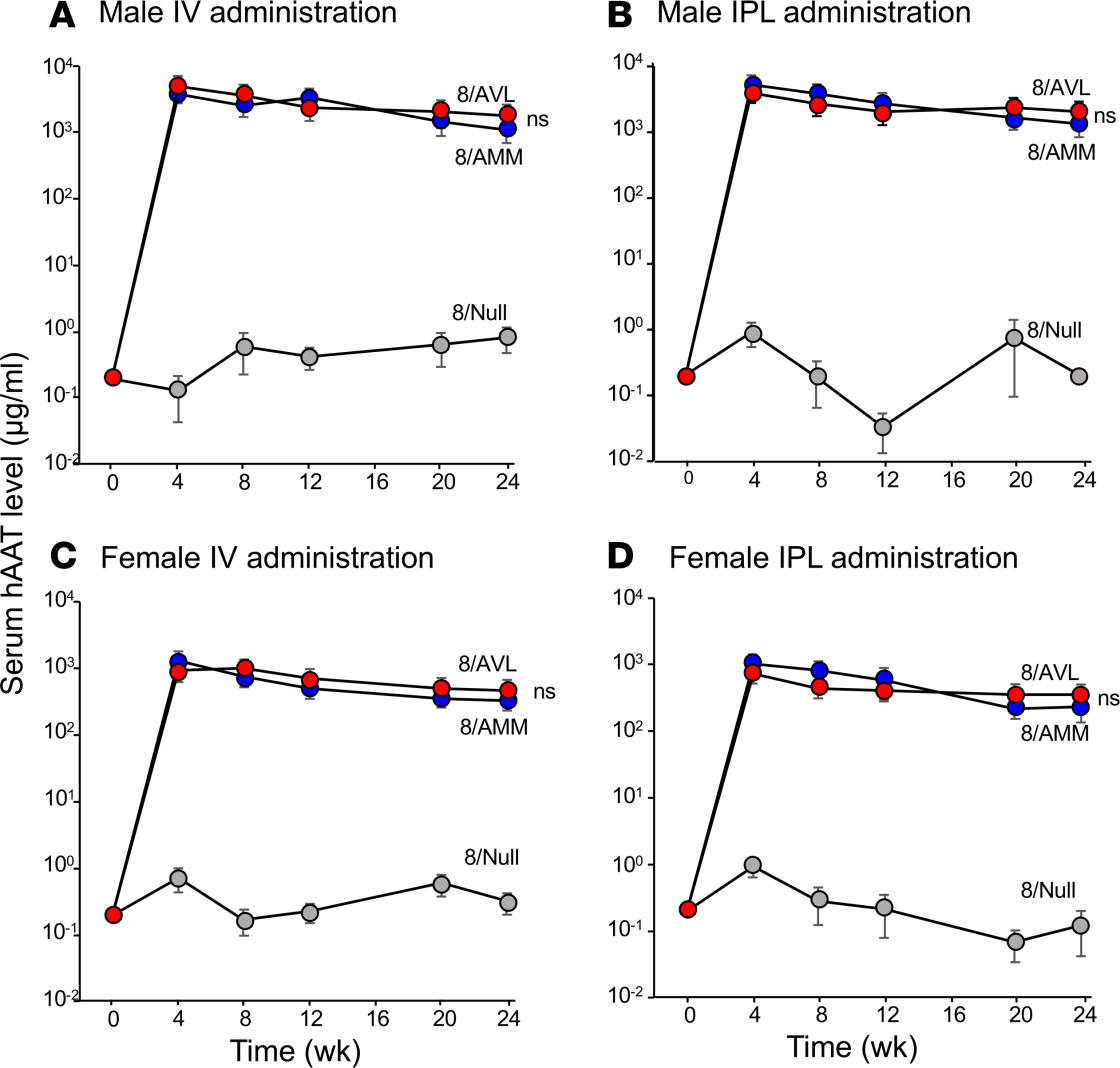
In vivo levels of human AAT variants in serum over time following IV or IPL administration in male and female mice (*n* = 4–5/group). C57BL/6 mice were administered 8/AMM, 8/AVL, or 8/Null (4 × 10^11^ gc) by IV and IPL routes. Serum was collected at 0, 4, 8, 12, 20, and 24 weeks, and human AAT was quantified by ELISA. (**A**) Male mice, IV. (**B**) Male mice, IPL. (**C**) Female mice, IV. (**D**) Female mice, IPL. At 24 weeks, there was no significant (ns) difference between 8/AMM and 8/AVL for either sex or administration route using Student’s *t* test.

Because of the superior ability of AAT-AVL to inhibit NE and cathepsin G under oxidizing conditions, we hypothesized that a onetime IV administration of 8/AVL would provide high levels of AAT in the serum and lung ELF that could effectively inhibit NE under oxidizing conditions. Based on prior studies demonstrating that intrapleural (IPL) administration yielded higher lung ELF levels (55), IPL administration was compared with IV administration. To evaluate this hypothesis, we administered 4 × 10^10^, 10^11^, or 4 × 10^11^ gc of 8/AVL oxidation-resistant variant via IV and IPL routes to male and female 6- to 8-week-old C57BL/6 mice (*n* = 5/group). WT 8/AMM (4 × 10^11^ gc) and 8/Null (no transgene; 4 × 10^11^ gc) were used as controls. Vector DNA in the liver and serum and ELF human AAT levels and function were assessed at various time points. Vector DNA in the liver showed high, dose-dependent, sustained levels over 24 weeks for IV and IPL administration in male (Supplemental Figure 2, A and B) and female mice (Supplemental Figure 2, C and D). AAT levels in serum and ELF for both IV and IPL routes of administration showed dose-dependent increases (male: Supplemental Figure 3; female: Supplemental Figure 4). Quantification by ELISA demonstrated high levels of human AAT in serum from 8/AVL (4 × 10^11^ gc) that were comparable to those from 8/AMM through 24 weeks in male mice administered IV (Figure 5A; 24 weeks 8/AVL: 1826 ± 201 μg/mL; 8/AMM: 1117 ± 323 μg/mL) or IPL (Figure 5B; 24 weeks 8/AVL: 1785 ± 455 μg/mL; 8/AMM: 1557 ± 389 μg/mL). Similar results were found in female mice (4 × 10^11^ gc), although the overall levels of AAT from both 8/AVL and 8/AMM were 1 log lower (Figure 5, C and D). Sex differences in transgene levels have been described previously and are specific to mice (61, 83, 84).

**Figure 6:**
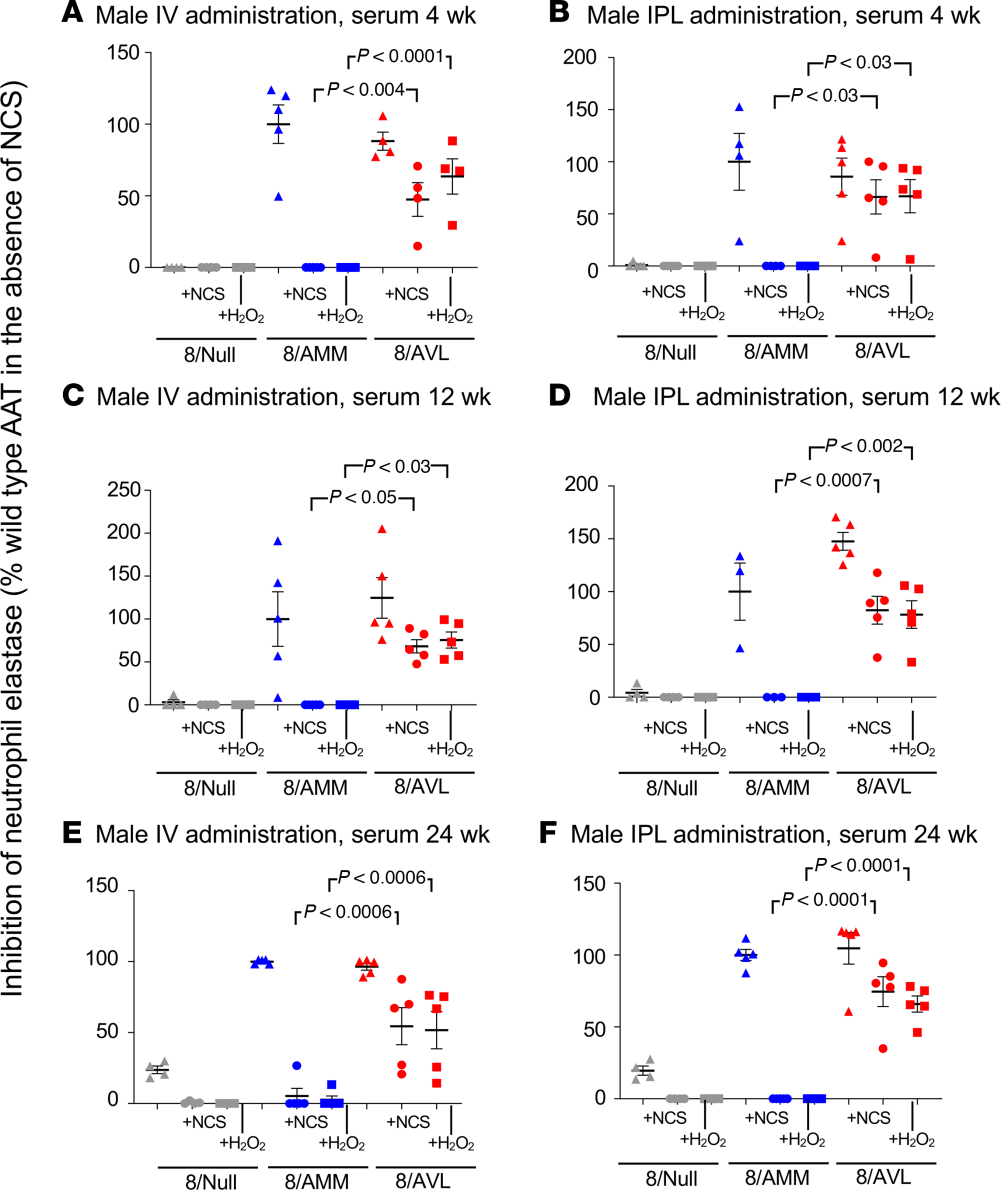
Anti-NE activity of human AAT in serum from male mice administered AAV8 vectors under normal and oxidizing conditions. C57BL/6 male mice were administered 8/AMM, 8/AVL, or 8/Null (4 × 10^11^ gc) by IV and IPL routes. Serum was collected at 4, 12, and 24 weeks, and human AAT was quantified by ELISA. Equal amounts (50 nM) of AAT were used in the NE inhibition assay. Data are presented as the percentage of WT (8/AMM) NE inhibition in the absence of oxidizer. (**A**) IV, 4 weeks. (**B**) IPL, 4 weeks. (**C**) IV, 12 weeks. (**D**) IPL, 12 weeks. (**E**) IV, 24 weeks. (**F**) IPL, 24 weeks. Assays were run in triplicate and averaged for samples from *n* = 4–5 mice/group, and statistical analysis was performed by ANOVA.

The function of AAT in serum from mice administered 8/AVL or 8/AMM (4 × 10^11^ gc) was examined using the NE inhibition assay at several time points. Serum from 8/AVL- and 8/AMM-administered male mice showed similar levels of NE inhibition in the absence of oxidizers at 4, 12, and 24 weeks for both IV and IPL routes (Figure 6, A–F). In the presence of oxidants NCS or H_2_O_2_, NE inhibition by AAT from 8/AMM-administered mice was abolished. Importantly, AAT from serum of 8/AVL-administered mice inhibited more than 50% of NE activity at all time points for both routes of administration, significantly higher than AAT from 8/AMM (all *P* < 0.03). Similar results were found with AAT from serum of female mice administered 8/AVL by either the IV or IPL routes (Supplemental Figure 5, A–F). Additionally, AAT in serum from male mice administered 8/AVL IV or IPL at 24 weeks was able to inhibit NE in the presence of hypochlorous acid (HOCl), an oxidant produced by activated neutrophils (85), while activity of AAT from 8/AMM-administered mice was abolished (Supplemental Figure 6, A and B).

**Figure 7:**
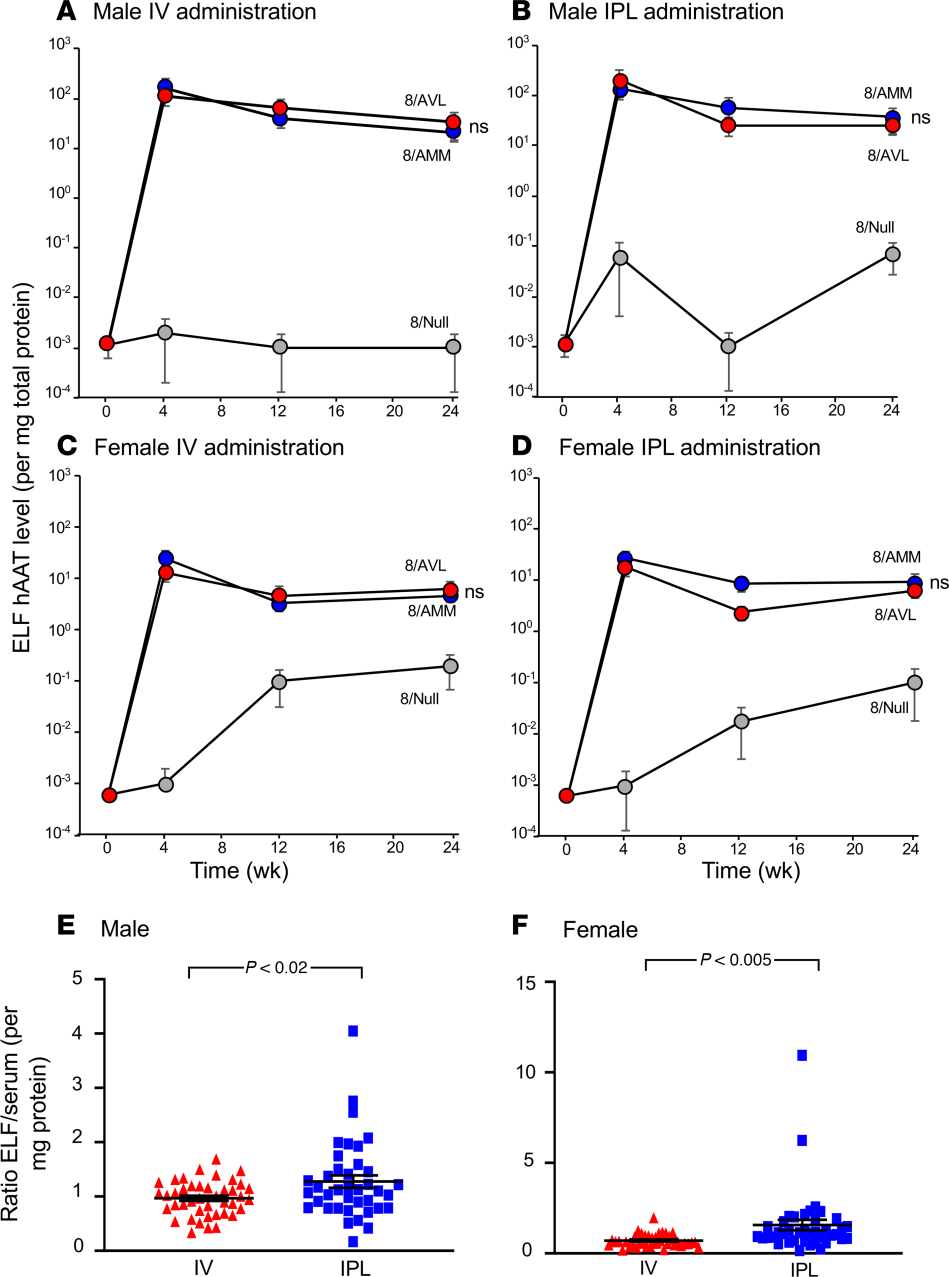
In vivo levels of human AAT in ELF over time following administration of the AAV8 vectors. C57BL/6 mice were administered 8/AMM, 8/AVL, or 8/Null (4 × 10^11^ gc) by the IV and IPL routes (*n* = 4–5/group). Lung ELF was collected after sacrifice at 0, 4, 12, or 24 weeks, and human AAT was quantified by ELISA. (**A**) Male mice, IV. (**B**) Male mice, IPL. (**C**) Female mice, IV. (**D**) Female mice, IPL. At 24 weeks, there was no significant (ns) difference between 8/AMM and 8/AVL for either sex or administration route using Student’s *t* test. (**E** and **F**) Ratio of ELF to serum AAT. (**E**) Male mice. (**F**) Female mice. For **E** and **F**, data from all doses and time points were combined, and AAT levels (per mg protein) were collectively compared in ELF and serum for mice administered 8/AVL. Statistical analysis was performed using Student’s *t* test.

**Figure 8:**
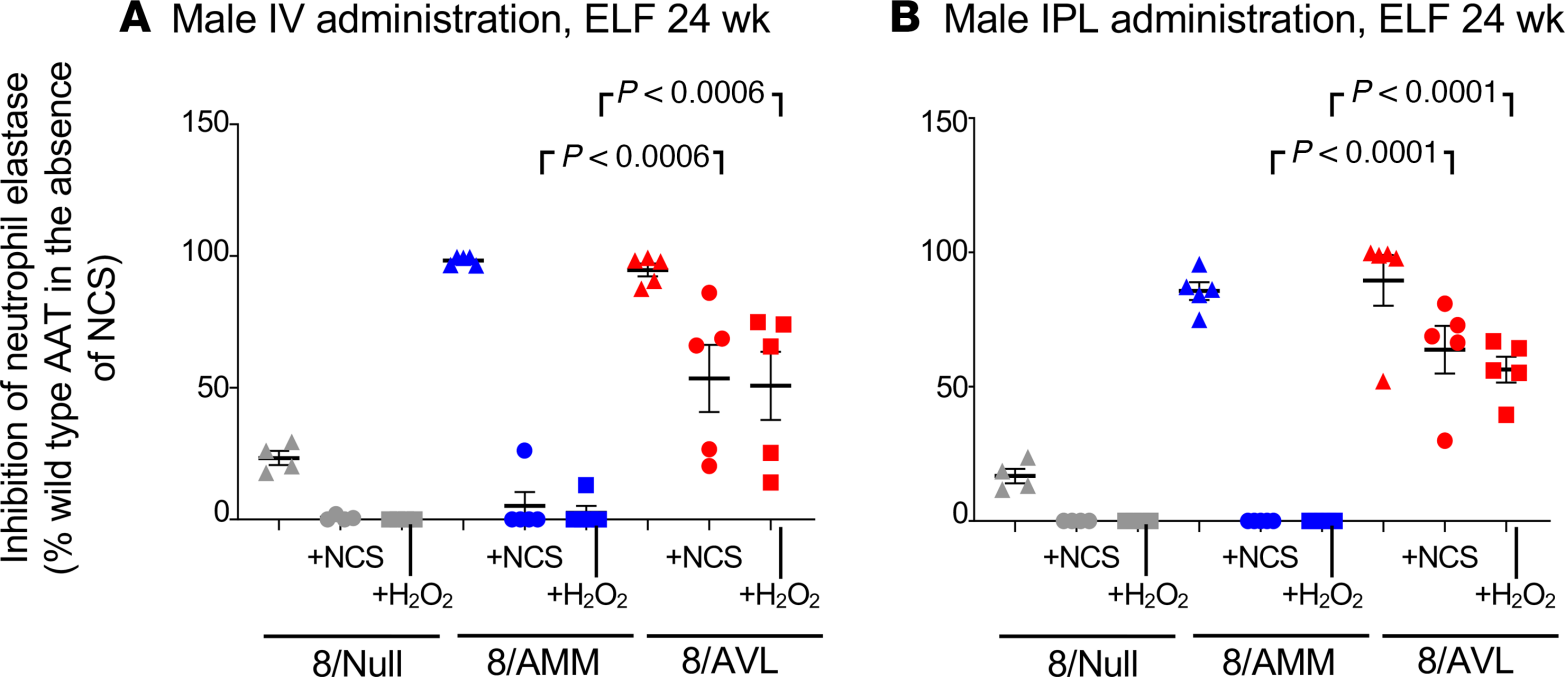
Anti-NE activity of human AAT in ELF from male mice administered AAV8 vectors under normal and oxidizing conditions. C57BL/6 male mice were administered 8/AMM, 8/AVL, or 8/Null (4 × 10^11^ gc) by IV and IPL routes. ELF was collected at 24 weeks and human AAT quantified by ELISA. Equal amounts (50 nM) of AAT were used in the NE inhibition assay. Data are presented as the percentage of WT (8/AMM) NE inhibition in the absence of oxidizer. Assays were run in triplicate and averaged for samples from *n* = 4–5 mice/group. Statistical analysis was performed by ANOVA. (**A**) IV, 24 weeks. (**B**) IPL, 24 weeks.

Human AAT levels in ELF of male mice after IV (Figure 7A) or IPL (Figure 7B) administration with 8/AVL (4 × 10^11^ gc) were 29.0 ± 2.6 μg/mg and 30.2 ± 4.7 μg/mg, respectively, at 24 weeks. These levels were similar to those of the mice administered 4 × 10^11^ gc 8/AMM (IV: 25.0 ± 7.2 μg/mg; IPL: 31.7 ± 6.3 μg/mg). Female mice also showed consistent levels of human AAT in the ELF after IV (Figure 7C) and IPL (Figure 7D) administration out to 24 weeks (4 × 10^11^ gc). When compared collectively across all doses and time points, the ratio of human AAT in the ELF/serum (per mg protein) after 8/AVL administration was significantly higher for the IPL administration compared with IV administration for both male and female mice (Figure 7, E and F). The ability of AAT in ELF to inhibit NE was assessed at 24 weeks. AAT from 8/AVL-administered mice retained significant inhibition of NE compared with AAT in ELF from 8/AMM administered either IV or IPL (Figure 8, A and B). Similar results were found with AAT from ELF of female mice administered 8/AVL by either the IV or IPL routes (Supplemental Figure 5, G and H).

## Discussion

AAT deficiency is a common hereditary disorder associated with early-onset emphysema and liver disease (1–3, 6–8). AAT, a serine protease inhibitor, functions to protect the lung from destruction by neutrophil-released proteases, such as NE (1, 2, 4, 6, 9, 10, 13). Greater than 95% of clinical cases of AAT deficiency result from low levels of AAT from the Z allele of the *SERPINA1* gene (1, 2, 4–6, 23, 26–28). The approved therapy for AAT deficiency is weekly infusions of purified AAT from pooled human plasma (9, 46–48). While this treatment is safe and effective, there is a high burden of cost as well as the requirement for repeated treatments for a lifetime. Further, AAT is susceptible to oxidant-mediated inactivation by both endogenous (inflammatory cells) and exogenous (cigarette smoke, pollution) oxidants, leaving it incapable of functioning as an antiprotease.

To overcome the shortcomings of the current treatments for AAT deficiency, we have developed an AAV-mediated gene therapy strategy using an AAV8 gene transfer vector coding for an oxidant-resistant AAT (8/AVL). The data demonstrate that administration of 8/AVL by either the IV or IPL route mediates high, persistent levels of AAT in the serum and ELF comparable to WT 8/AMM for at least 6 months. Importantly, AAT in serum or ELF from mice administered 8/AVL inhibited serine proteases even when exposed to an excess of oxidizing agents. 8/AVL gene therapy provides an AAT shield that will protect against serine proteases even in a lung environment persistently exposed to oxidants.

### Oxidation of AAT in vivo.

The lungs are constantly exposed to oxidizing agents both from endogenous sources, such as activated inflammatory cells, and exogenous sources, including air pollutants and cigarette smoke (86, 87). These oxidants inactivate AAT, making it incapable of inhibiting NE, cathepsin G, and other serine proteases that, if left unchecked, are destructive to the fragile alveolar structures. AAT recovered from the lungs of smokers has markedly lower functional activity than AAT recovered from nonsmokers (34). Further, alveolar macrophages recovered from the lungs of smokers spontaneously release greater superoxide and H_2_O_2_ than macrophages from nonsmokers, causing a 60% reduction in the ability of AAT to inhibit NE (88). Nonsmokers are still exposed to exogenous oxidants, such as pollutants, and thus there is likely a variable fraction of AAT in ELF that is actually active and able to inhibit proteases. Children with the ZZ genotype at high risk for developing lung disease show higher levels of oxidative stress markers in serum than children with the MM or MZ genotype (89). Thus, the susceptibility to oxidants likely contributes to the pathogenesis of emphysema in AAT-deficient individuals (1–3, 6–8).

### Gene therapy for AAT deficiency.

Gene therapy for AAT deficiency offers the potential for a onetime administration to provide therapeutic levels of AAT to protect the lung from destruction associated with the imbalance of proteases to antiproteases when AAT levels are low. Several strategies for gene therapy for AAT have been tested at the preclinical and clinical levels, but all have focused on delivering the normal human AAT M allele (55–73). The threshold level of AAT needed to be protective clinically is 11 μM in serum and 1.2 μM in ELF (2, 25, 47, 54). While these levels have not been achieved in AAT gene therapy clinical trials to date (59, 60, 63, 68), these calculations are based on WT AAT, a portion of which may be inactivated by oxidation. Therapy with the oxidant-resistant 8/AVL may prove efficacious in alleviating disease at lower absolute levels of AAT protein because of the elevated percentage of antiprotease activity, and lower vector doses may be needed to achieve this protein level.

### Protein therapy for AAT deficiency with oxidant-resistant AAT.

Protein therapy studies support the proposed use of oxidant-resistant AAT as a therapy for AAT deficiency. Early studies showed that an active, nonglycosylated human AAT containing the M358V mutation produced in yeast was able to inhibit NE under a number of oxidizing conditions in vitro (74, 78, 82). Similarly, recombinant human AAT with M358V, M358L, or M351V/M358V modifications produced in *Escherichia coli* showed oxidant resistance (75, 82, 90). Human AAT M358V produced in the plant *Nicotiana benthamiana* maintained antiprotease activity in the presence of oxidants compared with WT human AAT (80). Recombinant AAT fused to insulin-like growth factor produced in baculovirus with the M351 changed to glutamate (M351E) was a more potent inhibitor of NE than WT after incubation at room temperature for 24 hours (91). Recently, an oxidant-resistant recombinant human AAT-Fc fusion protein with M351E and M358L mutations (INBRX-101) was approved for testing in a phase I clinical study (81) (ClinicalTrials.gov NCT03815396). However, all of these oxidant-resistant AATs are recombinant proteins that would still need to be infused frequently, similar to the currently approved therapies (9, 46–48).

### IV versus IPL routes of administration.

Administration of 8/AVL by both the IV and IPL routes showed high levels of sustained AAT protein in the serum and ELF of both male and female mice. However, analyzing all doses and time points collectively, the ELF/serum ratio of AAT was significantly higher with the IPL administration route (Figure 7, E and F), suggesting that, for the same dose of the AAV vector, the IPL route may be more effective at defending the lung than IV administration. In addition to the mesothelial lining of the pleura as a site for gene expression directly in the lung, the parietal pleura has open stomata for lymphatics that allow for a slow release of the vector into the circulation, with consequent liver expression (92, 93). Thus, the IPL route provides for both local expression in the lung as well as expression from hepatocytes, which normally produce the majority of functional AAT (1, 2, 4, 6, 14–16, 46). The IPL route was shown to be safe in AAV gene therapy nonhuman primate studies (58, 94). Additionally, IPL administration delivers 8/AVL locally to a site where inflammation is unlikely to impede lung function, bypassing the respiratory epithelial route with its myriad of barriers to viral entry (95–97). IPL administration of the second-generation oxidant-resistant AAT-AVL protein expressed by the 8/AVL vector may be the ideal approach to treat AAT deficiency.

## Methods

### Initial testing of oxidant-resistant AAT constructs.

To identify the optimal configuration of substitutions in the human AAT coding sequence at M351 and M358, site-directed mutagenesis was used to modify the 351 and 358 residues to V or L in the common, normal M1(A213) allele. We chose the M1(A213) allele as the base allele because the Z mutation is derived from M1(A213), so the use of the same base may minimize the potential for immune system identification of a different residue in the 213 position (e.g., V213, another common normal M1 variant; Figure 1A). To make the various combinations of 351 and 358 modifications, QuikChange II Site-Directed Mutagenesis Kit (Agilent Technologies) was used with a modified protocol. The list of the variants at 351 and 358 is in Figure 1B. Briefly, 50 ng of template DNA was mixed with 125 ng of both forward and reverse primers for desired modification, DMSO, and 2x PfuUltra Hotstart PCR Master Mix (Agilent Technologies). Each reaction was then diluted to a final volume of 50 μL with molecular grade water (Corning Life Sciences). All primers used to modify nucleotides in the template human M1(V213) AAT cDNA were from MilliporeSigma (sequences listed in Supplemental Table 1). PCR reactions were mixed on ice and amplified for 18 cycles (95°C for 50 seconds, 60°C for 50 seconds, and 68°C for 16 minutes). The PCR reaction was digested by DpnI (New England BioLabs) for 1 hour at 37°C to degrade template DNA. Amplified DNA was precipitated using ethanol before transforming MAX Efficiency DH5α competent cells obtained from Invitrogen, Thermo Fisher Scientific, according to the manufacturer’s instructions. Transformed cells were spread on LB agar plates supplemented with 50 μg/mL kanamycin (Teknova) and incubated overnight at 37°C. Colonies were inoculated into LB broth supplemented with 50 μg/mL kanamycin shaking cultures overnight at 37°C. Plasmid DNA was purified according to the manufacturer’s instructions using the QIAprep Spin Miniprep Kit (QIAGEN). All constructs were sequenced (Genewiz) to confirm the mutations in respective plasmids. Finally, all correctly modified variants were subcloned into the AAV expression cassette containing the CMV enhancer, chicken β-actin promoter, splice donor and intron, and rabbit β-globin splice acceptor (CAG), with the modified human AAT cDNA and rabbit β-globin poly(A) site flanked by the inverted terminal repeats from AAV2 (Supplemental Figure 1).

### Assessment of plasmid constructs.

Human embryonic kidney 293T cells (American Type Culture Collection) were maintained in Dulbecco’s modified Eagle medium (Corning Cellgro) supplemented with 10% fetal bovine serum (Life Technologies, Thermo Fisher Scientific), 1 mM glutamine, 1 mM sodium pyruvate, and 50 μg/mL gentamicin. To assess expression of the modified AAT proteins in vitro, 293T cells were transfected with 3 μg modified AAT plasmid per well and harvested at 72 hours postinfection. Media from untransfected cells served as negative “mock” controls. The media were evaluated for the expression of the human AAT after concentration of sample volume using Amicon Ultra centrifugal filters (MilliporeSigma) with 10 kDa cutoff.

The levels of human AAT in transfection media were determined using an ELISA kit specific for human AAT (American Laboratory Products Company; ALPCO catalog 30-6752) and a highly purified AAT standard (courtesy of M. Brantly, University of Florida, Gainesville, Florida, USA).

### Serine protease inhibition assays.

To compare the biological activity of the AAT constructs, inhibition of NE activity was assessed using N-Suc-Ala-Ala-Ala-*p*-nitroanilide substrate following the manufacturer’s protocol (Elastin Products Co.). All assays were performed in 100 μL of Tris-NaCl buffer (0.1 M Tris pH 7.5, 0.5 M NaCl) in a 96-well plate. Human NE (0.1 μg, Elastin Products) was resuspended in 0.05 M sodium acetate (NaOAc), pH 5, with 0.1 M NaCl and kept on ice until use. For all oxidation studies, 50 nM AAT was preincubated with 1 mM NCS (MilliporeSigma) for 20 minutes, 250 mM H_2_O_2_ (MilliporeSigma) for 40 minutes, or 200 μM HOCl (MilliporeSigma) for 30 minutes before addition of 200 nM NE final concentration for 5 minutes. Substrate (2 mM N-Suc-Ala-Ala-Ala-*p*-nitroanilide) was then added, and initial reaction rates were measured by monitoring change in absorbance at 410 nm for 1 hour using the SpectraMax i3 plate reader (Molecular Devices, Thermo Fisher Scientific). Oxidants were not removed before addition of substrate (36, 38, 76, 78).

The assay for cathepsin G activity was carried out using N-Suc-Ala-Ala-Pro-Phe-*p*-nitroanilide substrate following the manufacturer’s protocol (Elastin Products). All assays were carried out in 100 μL of Tris-NaCl buffer (0.1 M Tris pH 7.5, 0.5 M NaCl) in a 96-well plate. Cathepsin G (0.1 μg, Elastin Products) was resuspended in 0.05 M NaOAc, pH 5, with 0.1 M NaCl and kept on ice until use. For all oxidation studies, 200 nM AAT was preincubated with 1 mM NCS (MilliporeSigma) for 20 minutes or 250 mM H_2_O_2_ (MilliporeSigma) for 40 minutes before addition of 200 nM cathepsin G final concentration for 5 minutes. Substrate (2 mM N-Suc-Ala-Ala-Pro-Phe-*p*-nitroanilide) was then added, and initial reaction rates were measured by monitoring change in absorbance at 410 nm for 1 hour using the SpectraMax i3 plate reader (Molecular Devices, Thermo Fisher Scientific). Oxidants were not removed before addition of substrate (40).

### Association rate studies.

Association rate studies of the AAT variants’ interaction with NE were carried out with purified AAT-AMM and AAT-AVL. 293T cells were transfected with AAT plasmids, and media were harvested 72 hours posttransfection as described above. The AAT was purified using Alpha-1 Antitrypsin Select resin (17547201, GE Healthcare) according to the manufacturer’s instructions. Following elution, purified AAT proteins were dialyzed into PBS pH 7.4, concentrated using Amicon Ultra spin concentrators with 10 kDa cutoff (MilliporeSigma), and quantified by ELISA (ALPCO). The association rate studies were performed by surface plasmon resonance on the Bio-Rad ProteOn XPR36 Protein Interaction Array System (Bio-Rad Laboratories) at the High Throughput Screening Resource Center at the Rockefeller University using a GLC sensor chip consisting of a 6 × 6 sample channel matrix. After initialization and conditioning according to the manufacturer’s instructions, channel 1 of the GLC chip was cleaned sequentially with 0.5% sodium dodecyl sulfate, 50 mM NaOH, and 100 mM HCl. The channel was then activated for amine coupling with 20 mM 1-ethyl-3-(3-dimethylaminopropyl) carbodiimide hydrochloride and 5 mM N-hydroxysulfosuccinimide (ProteOn Amine Coupling Kit, Bio-Rad Laboratories). NE was diluted to 10 μg/mL in 50 mM acetate buffer, pH 5.5, and flowed across the sensor chip to allow binding, and the response units were recorded. The chip was quenched with 1 M ethanolamine pH 8.5. The running buffer during NE-binding steps was 10 mM HEPES, 150 mM NaCl, and 0.01% Tween-20. After completion of NE binding, the running buffer was switched to 10 mM HEPES, 150 mM NaCl, 0.01% Tween-20, and 2 mg/mL bovine serum albumin (BSA binding buffer). Purified AAT was diluted to 1000 nM in BSA binding buffer, and 2-fold serial dilutions were made in BSA binding buffer to 62.5 nM. Buffer alone was used as a blank. Each AAT dilution, in separate lanes (1–6), was flowed simultaneously across the NE-bound channel for 60 seconds to allow association to occur, with the wash delay set for 600 seconds to allow for dissociation. The *K_a_* was calculated by ProteOn Manager software (Bio-Rad Laboratories) using the kinetic Langmuir binding model with simultaneous calculation of binding association and dissociation rates (98). The process was repeated as described for each AAT variant and condition starting with binding NE to a new channel for each experiment because the interaction between NE and AAT is irreversible. For binding under oxidizing conditions, AAT purified protein was incubated with 1 mM NCS for 20 minutes before dilution in BSA binding buffer. The running buffer for experiments under oxidizing conditions was 10 mM HEPES, 150 mM NaCl, 0.01% Tween-20, 2 mg/mL BSA, and 4.75 μM NCS. In all cases, the χ^2^ value for goodness of fit was less than 10% of R_max_, indicating that the data were a good fit for the binding model.

### AAV8-based gene transfer vectors.

The AAV8 vectors were produced using 293T cells with a 3-plasmid transfection system as described previously (61). Briefly, the pAAV-CAG-hAAT expression plasmid (55 μg), AAV8 packaging plasmid containing Rep (derived from AAV2) and Cap (derived from AAV8) proteins (550 μg), and pAdDeltaF6 adenovirus helper plasmid (1100 μg) were cotransfected into 293T cells using PEI transfection reagent (Polysciences). At 72 hours posttransfection, cells were harvested and lysate prepared using 5 cycles of freeze/thaw. Cell lysate containing the virus was clarified by centrifugation at 2675 *g* for 15 minutes. The AAV8 vectors were purified from the crude viral lysate by iodixanol gradient. Vectors were diluted in PBS, pH 7.4, with 5% glycerol and 35 mM NaCl. Iodixanol was removed by dialysis using Slide-A-Lyzer Dialysis cassettes (12–30 mL, 20 kDa cutoff; Thermo Fisher Scientific). Vectors were concentrated using a Vivaspin 20 100K membrane concentrator (GE Healthcare) and stored in PBS, 5% glycerol, and 35 mM NaCl at –80°C.

### IV and IPL vector delivery.

8/AMM, 8/AVL, and 8/null vectors (4 × 10^10^, 10^11^, 4 × 10^11^ gc) were delivered either IV or IPL to the left lung of 6- to 8-week-old C57BL/6 mice (*n* = 4 to 5) (61). For the IPL administration, the mice were anesthetized in the right lateral position with a combination of isoflurane (1% to 3% inhalation), buprenorphine (0.1 mg/kg subcutaneously), and bupivacaine (2 mg/kg, subcutaneously). An anterolateral chest skin incision approximately 1 cm in length was made using scissors and the thoracic cage was exposed. The third rib was lifted with tweezers, and the IPL injection via the third intercostal space was performed using a 1 mL insulin syringe. All doses of AAV vectors were administered in 100 μL of PBS. The skin was closed with 4-0 absorbable suture (Polysorb). Sutures were removed within 2 weeks. For IV delivery, AAV vectors were administered in 100 μL of PBS via the tail vein using a 1 mL insulin syringe.

### Sample collection.

Serum was collected at 0, 4, 8, 12, 20, and 24 weeks by drawing 200 μL of blood from the tail vein in BD Microtainer SST tubes (Becton Dickinson). The blood samples were allowed to clot for 10 minutes at 23°C and then spun in a microcentrifuge at 2000 *g* for 10 minutes at 4°C to collect serum. Mice were euthanized at weeks 4, 12, or 24 after vector administration using CO_2_ inhalation followed by cardiac perfusion with 40 mL cold PBS, pH 7.4. Livers were collected and immediately frozen on dry ice.

Immediately after being euthanized by CO_2_, mice were fixed in the supine position. The diaphragm was cut open and trachea exposed. The anterior wall of the trachea was cut, a 23-G angiocatheter was inserted, and the orifice was tied with 4-0 silk, including the inserted angiocatheter to keep the airway closed. Five hundred microliters PBS was injected and flushed twice and then collected. Lavage samples were kept on ice until processing by centrifugation at 10,000 *g* for 5 minutes at 4°C to remove any cells, and then supernatant was stored at –80°C (99).

### Analysis of serum and ELF.

Serum and ELF collected from mice at various time points were analyzed for human AAT levels by ELISA (ALPCO). Total protein in ELF was determined using the Pierce BCA Protein Assay Kit (Thermo Fisher Scientific). Inhibitory capacity of AAT in serum and ELF was assessed under normal and oxidizing conditions using the NE and cathepsin G inhibition assays as described above.

### Vector genome DNA analysis.

Vector genome DNA levels were assessed in the liver to evaluate persistence. Livers were weighed and homogenized in 1.5 mL of lysis buffer containing 10 mM HEPES KOH, pH 7.4; 5 mM mannitol; and 1% Triton X-100 per liver using 2.5 mm stainless steel beads in a TissueLyzer LT (QIAGEN) until no visible chunks remained (5 minutes at a time) at an oscillation of 50/s (100). The homogenate was collected, pooled, and stored at –80°C. Genomic DNA was isolated from the liver homogenate using the DNeasy Blood and Tissue kit (QIAGEN) from 200 μL of homogenate according to the manufacturer’s instructions. The total amount of DNA extracted was measured using a UV spectrophotometer (NanoDrop ND-1000, Thermo Fisher Scientific). The human AAT cDNA transgene contains silent changes in the last 200 nucleotides for detection by quantitative polymerase chain reaction (qPCR) (58). Human AAT gene expression was quantified by TaqMan qPCR with a FAM-labeled transgene specific primer/probe set (4331348, Applied Biosystems, Thermo Fisher Scientific) (58). Using relative quantification, 500 ng/well from the total DNA extracted was assayed and compared with a standard curve created by pAAV-CAG-hAAT plasmid that spanned the range of 10^2^–10^9^ gc per well (slope –3.48; *r*^2^ = 0.999). The efficiency of DNA extraction was measured by using a VIC-labeled housekeeping murine *Tfrc* gene specific primer/probe set (4458366, Applied Biosystems, Thermo Fisher Scientific) and compared with a predetermined specification (Ct value of 28.3 ± 4.3).

### Statistics.

All data are expressed as either geometric means (for serum titers) or group means (AAT levels) ± SEM. Comparisons for experimental groups were performed with 2-tailed Student’s *t* test and 1-way ANOVA for multiple comparisons within data sets.

### Study approval.

All mouse experiments conformed to the relevant regulatory standards and were approved by the Institutional Animal Care and Use Committee at Weill Cornell Medical College (WCMC 2014-0054).

## Author contributions

MLS and KMS designed and performed experiments, analyzed data, and wrote the manuscript; EZF, FMH, and YM performed experiments; BPD and SMK analyzed data; and RGC designed experiments, analyzed data, and wrote the manuscript.

## Supplementary Material

Supplemental data

## Figures and Tables

**Table 1:**
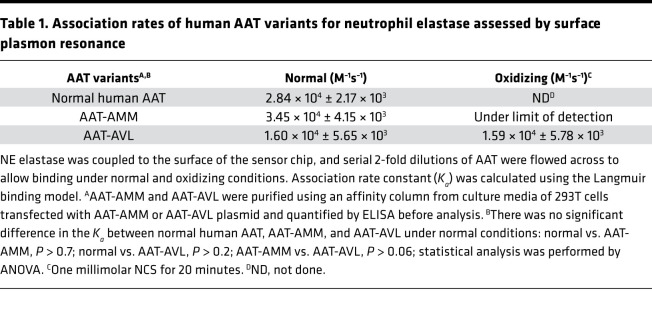
Association rates of human AAT variants for neutrophil elastase assessed by surface plasmon resonance

## Version 1 08/06/2020

Electronic publication
