# Supplementary material for: Gene therapy for alpha 1-antitrypsin deficiency with an oxidant-resistant human alpha 1-antitrypsin
Source: JCI Insight. 2020 Aug 6;5(15):e135951. doi: 10.1172/jci.insight.135951 (PMC7455074; doi:10.1172/jci.insight.135951)
Supplement: Supplemental data [file jciinsight-5-135951-s058.pdf]

Supplemental Table I. Sequences of Primers for Mutagenesis of Residues 213, 351 and 358

Modification	Sequence
Val213Ala	Forward 5’-CCACGTGGACCAGGCCACCACCGTGAAGGT-3’
	Reverse 5’-ACCTTCACGGTGGTGGCCTGGTCCACGTGG-3’
Met351Val	Forward 5’-GCAGCTGGCGCGGTGTTCTTGGAGGCGATC-3’
	Reverse 5’-GATCGCCTCCAAGAACACCGCGCCACGTGC-3’
Met351Leu	Forward 5’-CAGCTGGCGCGCTGTTCTTGGAGGCGATCC-3’
	Reverse 5’-GGATCGCCTCCAAGAACAGCGCGCCACGTG-3’
Met358Val	Forward 5’-CTTGGAGGCGATCCCAGTGTCGATCCCG-3’
	Reverse 5’-CGGGATCGACACTGGGATCGCCTCCAAG-3’
Met358Leu	Forward 5’-CTTGGAGGCGATCCCACTGTCGATCCCG-3’
	Reverse 5’-CGGGATCGACAGTGGGATCGCCTCCAAG-3’

Supplemental Figure Legends

Supplemental Figure 1. Schematic of the AAV8/AAT vectors. The expression cassette including the AAV2 inverted terminal repeats (ITR), CAG promoter, optimized human AAT variant cDNA, and polyadenylation signal (A_n) was packaged in the AAV8 capsid.

Supplemental Figure 2. *In vivo* levels of AAV vector derived DNA in liver as a function of time. C57Bl/6 male and female mice (n=4-5/group) were administered 8/AVL [$4x10^{10}$, 10^{11}, $4x10^{11}$ genome copies (gc)], 8/AMM ($4x10^{11}$ gc), or 8/Null ($4x10^{11}$ gc) by the intravenous (IV) or intrapleural (IPL) routes. At 0, 4, 12, 24 wk post-administration, mice were sacrificed and livers were harvested. DNA was extracted and vector DNA was quantified by Taqman qPCR. **A.** Males, IV administration. **B.** Males, IPL administration. **C.** Females, IV administration. **D.** Females IPL administration.

Supplemental Figure 3. Dose response of human AAT levels in serum and lung epithelial lining fluid (ELF) at 24 wk after 8/AVL administration in male mice. C57Bl/6 male mice (n=4-5/group) were administered 8/AVL ($4x10^{10}$, 10^{11}, $4x10^{11}$ gc), 8/AMM ($4x10^{11}$ gc), or 8/Null ($4x10^{11}$ gc) by the intravenous (IV) or intrapleural (IPL) routes. Serum and ELF was collected at 24 wk and human AAT was quantified by ELISA. Total protein in ELF was quantified by BCA assay. **A.** Serum 24 wk, IV. **B.** Serum 24 wk, IPL. **C.** ELF 24 wk, IV. **D.** ELF 24 wk, IPL.

Supplemental Figure 4. Dose response of human AAT levels in serum and epithelial lining fluid (ELF) at 24 wk after 8/AVL administration in female mice. C57Bl/6 female mice (n=4-5/group) were administered 8/AVL ($4x10^{10}$, 10^{11}, $4x10^{11}$ gc), 8/AMM ($4x10^{11}$ gc), or 8/Null ($4x10^{11}$ gc) by the intravenous (IV) or intrapleural (IPL) routes. Serum and ELF was collected at 24 wk and human AAT was quantified by ELISA. Total protein in ELF was quantified by BCA assay. **A.** Serum 24 wk, IV. **B.** Serum 24 wk, IPL. **C.** ELF 24 wk, IV. **D.** ELF 24 wk, IPL.

Supplemental Figure 5. Anti-NE activity of human AAT in serum and epithelial lining fluid (ELF) from female mice administered AAV8 vectors under normal and oxidizing conditions. C57Bl/6 female mice were administered 8/AMM, 8/AVL, or 8/Null ($4x10^{11}$ gc) by the intravenous (IV) or intrapleural (IPL) routes. Serum was collected at 4, 12, 24 wk and ELF at 24 wk. Human AAT was quantified by ELISA. Equal amounts (50 nM) of AAT were used in the NE inhibition assay. Data is presented as the % of wild-type (8/AMM) NE inhibition in the absence of oxidizer. Assays were run in triplicate and averaged for samples from n=4-5 mice/group. **A.** Serum, IV, 4 wk. **B.** Serum, IPL, 4 wk. **C.** Serum, IV, 12 wk. **D.** Serum, IPL, 12 wk. **E.** Serum, IV, 24 wk. **F.** Serum, IPL, 24 wk. **G.** ELF, IV, 24 wk. **H.** ELF, IPL, 24 wk.

Supplemental Figure 6. Anti-NE activity of human AAT in serum from male mice administered AAV8 vectors under normal and oxidizing conditions with hypochlorous acid (HOCl). C57Bl/6 male mice were administered 8/AMM or 8/AVL ($4x10^{11}$ gc) by intravenous (IV) and intrapleural (IPL) routes. Serum was collected at 24 wk and human AAT quantified by ELISA. Equal amounts (50 nM) of AAT were used in the NE inhibition assay. Data is presented as the % of wild-type (8/AMM) NE inhibition in the absence of oxidizer HOCl. Assays were run in triplicate and averaged for samples from n=4-5 mice per group. **A.** IV, 24 wk. **B.** IPL, 24 wk.

Figure S1

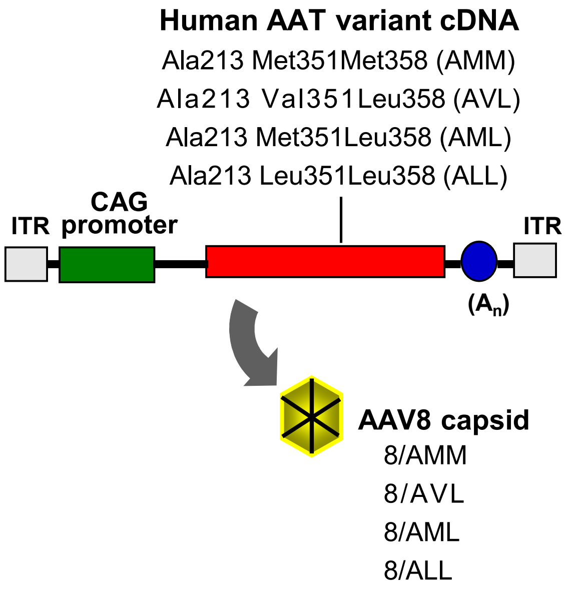

Human AAT variant cDNA
Ala213 Met351Met358 (AMM)
Ala213 Val351Leu358 (AVL)
Ala213 Met351Leu358 (AML)
Ala213 Leu351Leu358 (ALL)
ITR
CAG promoter
ITR
(An)
AAV8 capsid
8/AMM
8/AVL
8/AML
8/ALL

Figure S2

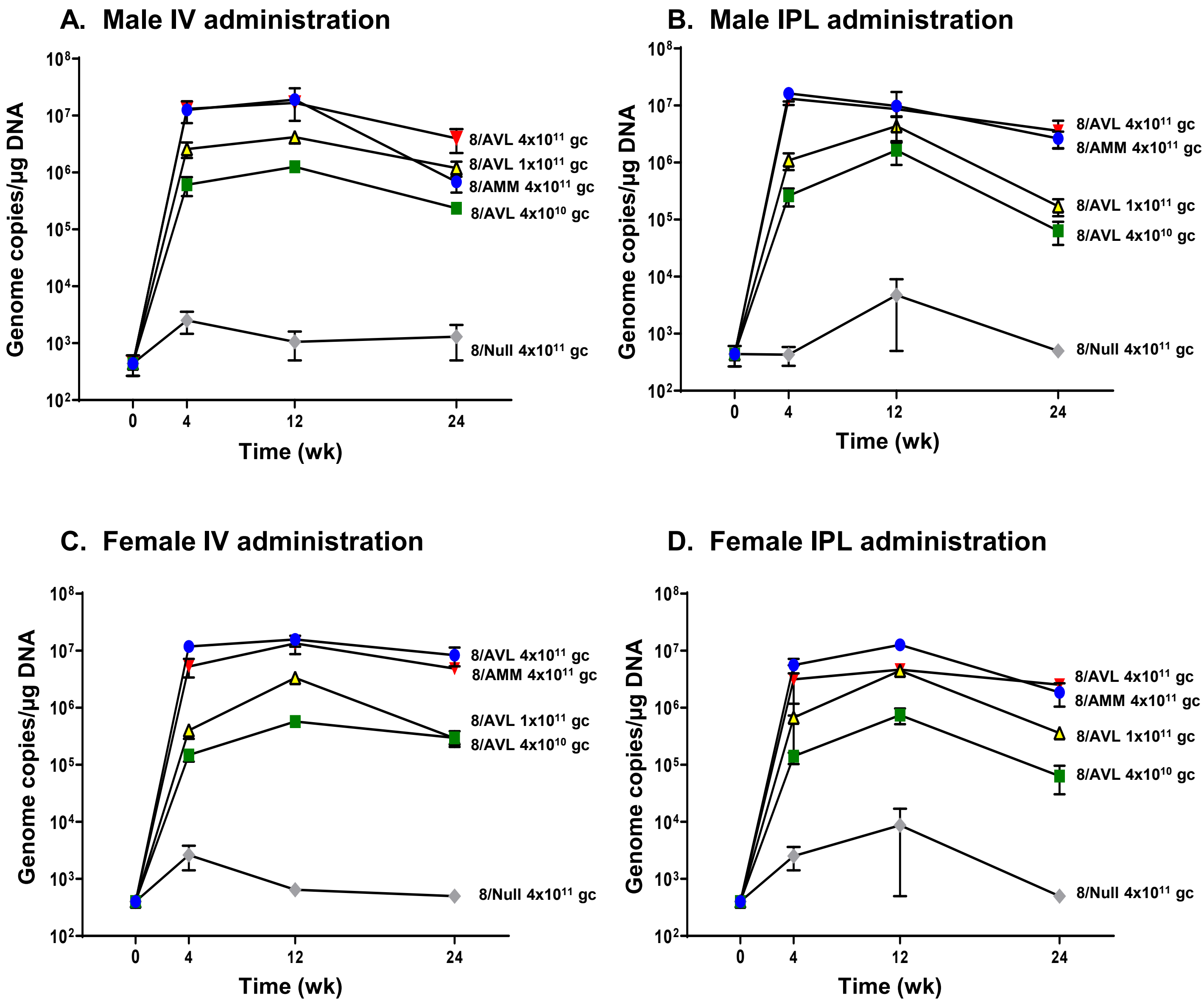
A. Male IV administration
Genome copies/µg DNA
Time (wk)
8/AVL 4x10¹¹ gc
8/AVL 1x10¹¹ gc
8/AMM 4x10¹¹ gc
8/AVL 4x10¹⁰ gc
8/Null 4x10¹¹ gc
B. Male IPL administration
Genome copies/µg DNA
Time (wk)
8/AVL 4x10¹¹ gc
8/AMM 4x10¹¹ gc
8/AVL 1x10¹¹ gc
8/AVL 4x10¹⁰ gc
8/Null 4x10¹¹ gc
C. Female IV administration
Genome copies/µg DNA
Time (wk)
8/AVL 4x10¹¹ gc
8/AMM 4x10¹¹ gc
8/AVL 1x10¹¹ gc
8/AVL 4x10¹⁰ gc
8/Null 4x10¹¹ gc
D. Female IPL administration
Genome copies/µg DNA
Time (wk)
8/AVL 4x10¹¹ gc
8/AMM 4x10¹¹ gc
8/AVL 1x10¹¹ gc
8/AVL 4x10¹⁰ gc
8/Null 4x10¹¹ gc

Figure S3

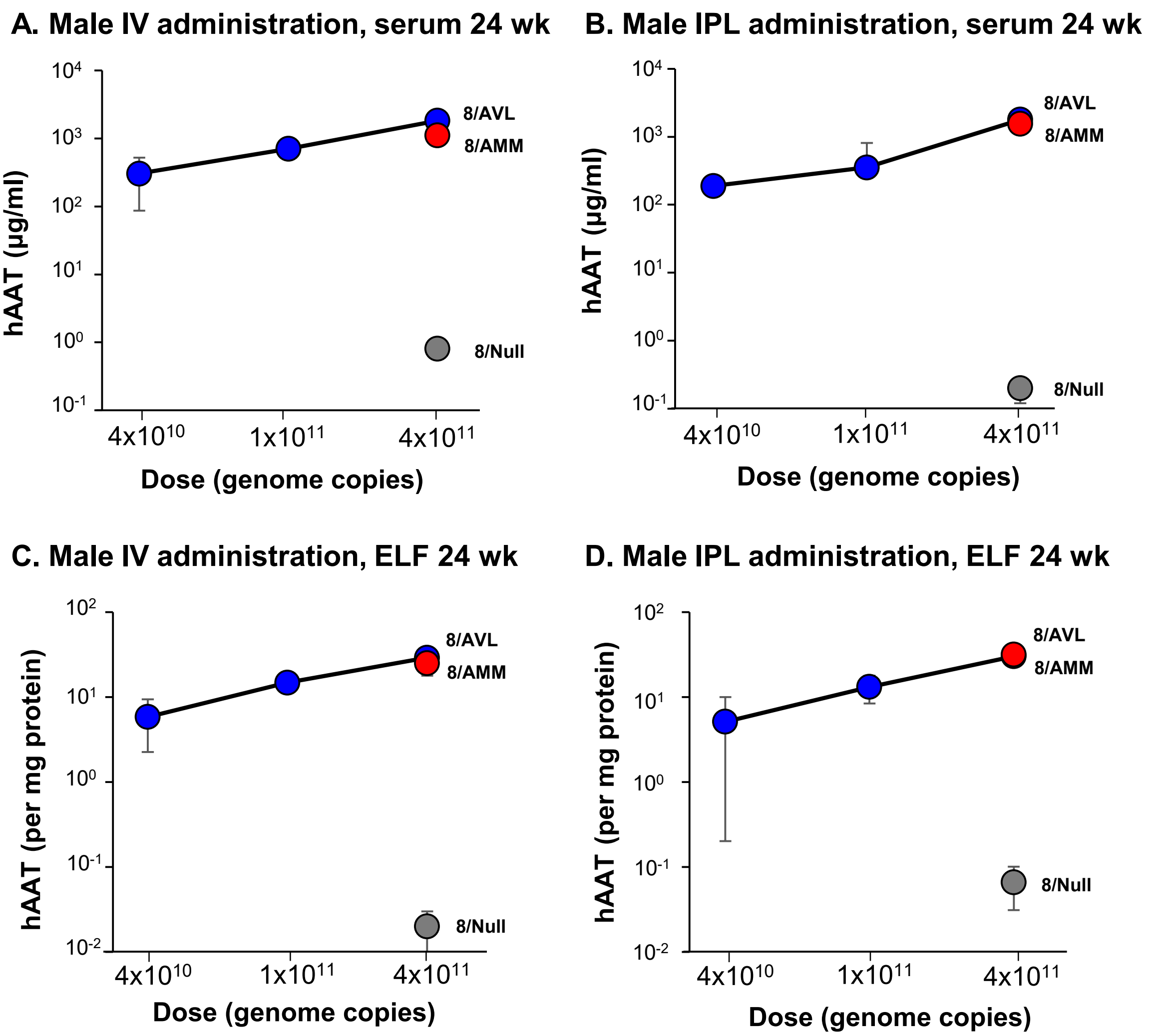

A. Male IV administration, serum 24 wk
hAAT (µg/ml)
10-1
10^0
10^1
10^2
10^3
10^4
8/AVL
8/AMM
8/Null
4x10^10
1x10^11
4x10^11
Dose (genome copies)
B. Male IPL administration, serum 24 wk
hAAT (µg/ml)
8/AVL
8/AMM
8/Null
Dose (genome copies)
C. Male IV administration, ELF 24 wk
hAAT (per mg protein)
10-2
10-1
10^0
10^1
10^2
8/AVL
8/AMM
8/Null
Dose (genome copies)
D. Male IPL administration, ELF 24 wk
hAAT (per mg protein)
8/AVL
8/AMM
8/Null
Dose (genome copies)

Figure S4

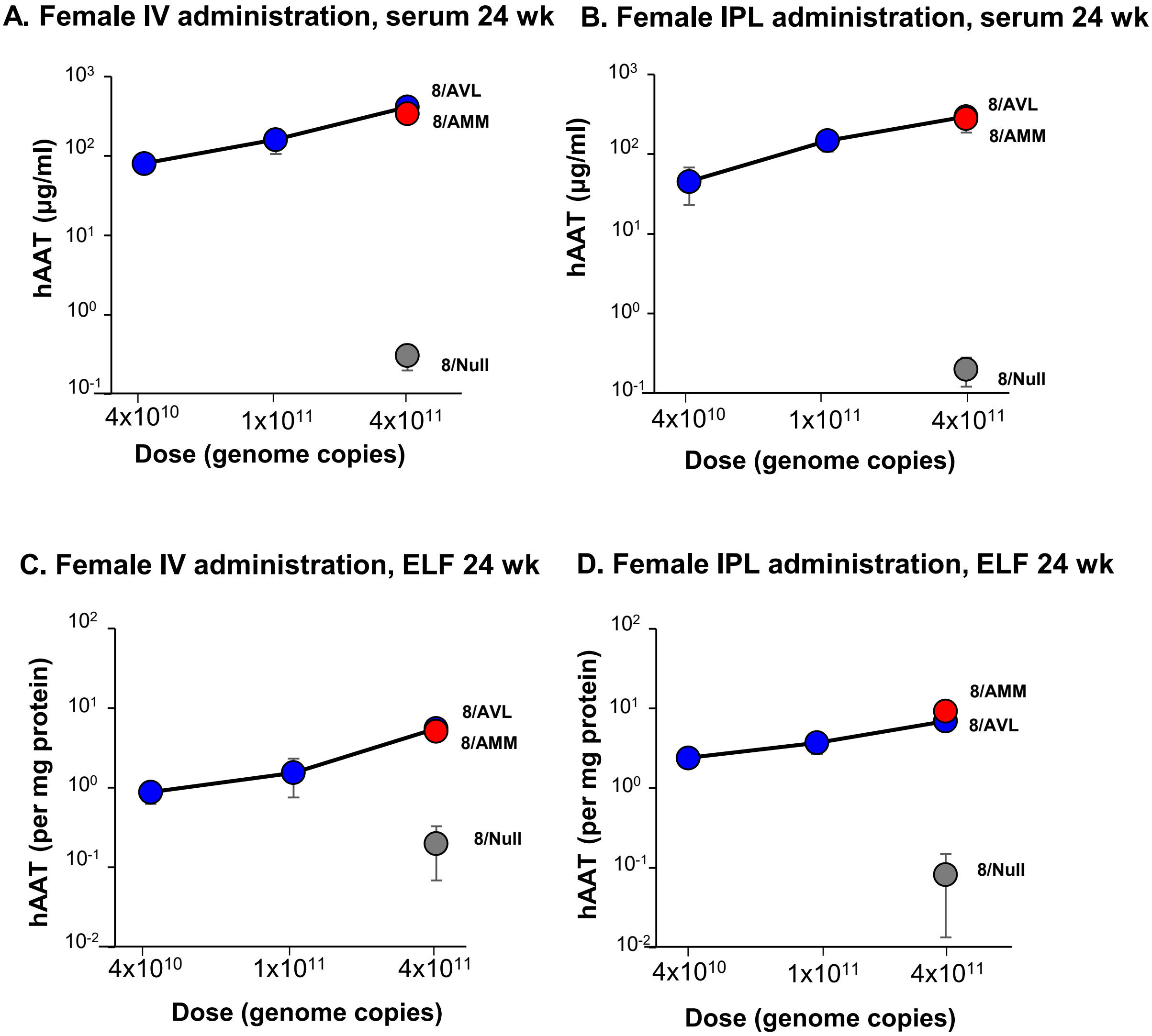

A. Female IV administration, serum 24 wk
hAAT (µg/ml)
8/AVL
8/AMM
8/Null
Dose (genome copies)
B. Female IPL administration, serum 24 wk
hAAT (µg/ml)
8/AVL
8/AMM
8/Null
Dose (genome copies)
C. Female IV administration, ELF 24 wk
hAAT (per mg protein)
8/AVL
8/AMM
8/Null
Dose (genome copies)
D. Female IPL administration, ELF 24 wk
hAAT (per mg protein)
8/AMM
8/AVL
8/Null
Dose (genome copies)

Figure S5

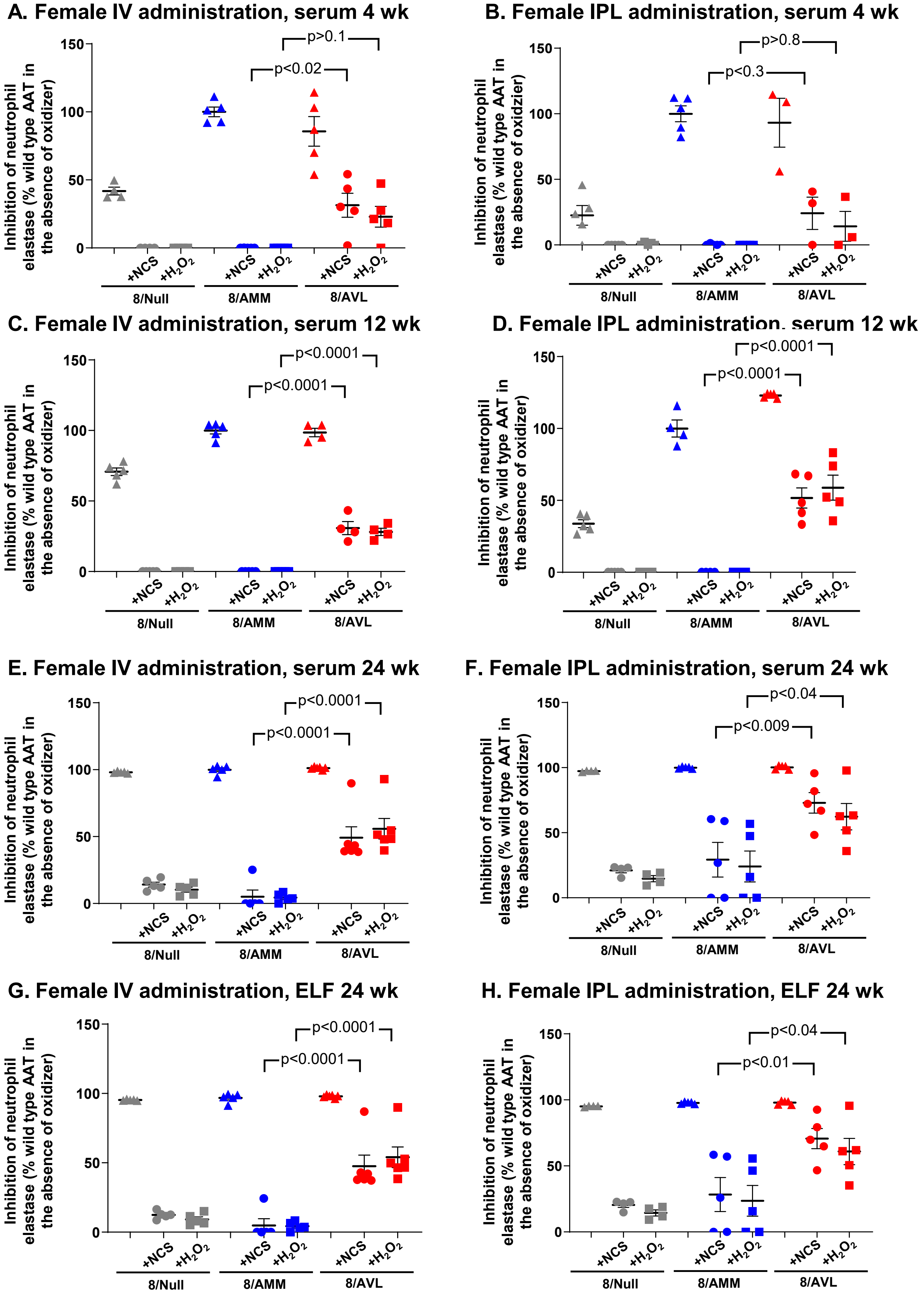

A. Female IV administration, serum 4 wk
B. Female IPL administration, serum 4 wk
C. Female IV administration, serum 12 wk
D. Female IPL administration, serum 12 wk
E. Female IV administration, serum 24 wk
F. Female IPL administration, serum 24 wk
G. Female IV administration, ELF 24 wk
H. Female IPL administration, ELF 24 wk
Inhibition of neutrophil elastase (% wild type AAT in the absence of oxidizer)
0
50
100
150
+NCS
+H2O2
8/Null
8/AMM
8/AVL
p>0.1
p<0.02
p>0.8
p<0.3
p<0.0001
p<0.04
p<0.009
p<0.01

Figure S6

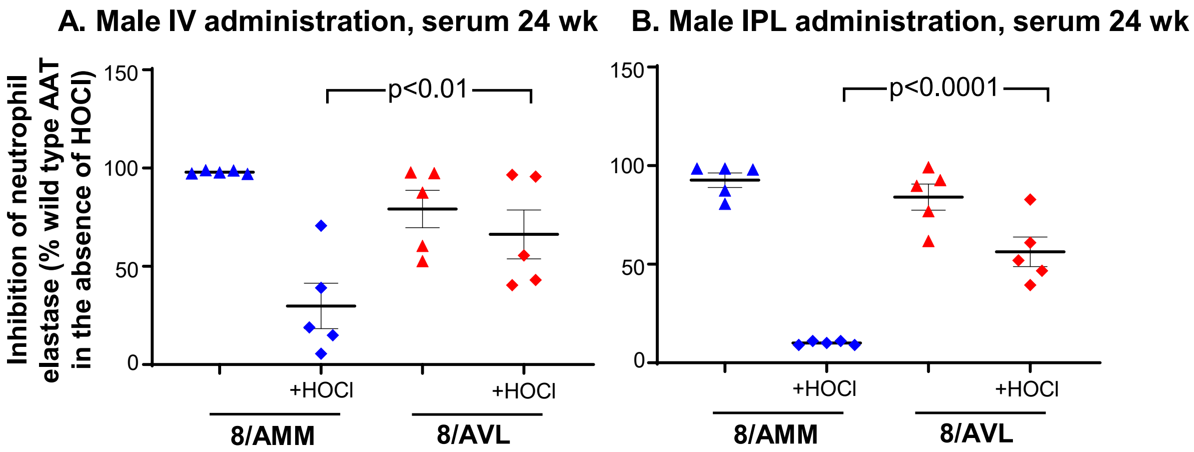

A. Male IV administration, serum 24 wk
B. Male IPL administration, serum 24 wk
Inhibition of neutrophil elastase (% wild type AAT in the absence of HOCl)
150
100
50
0
p<0.01
p<0.0001
+HOCl
+HOCl
8/AMM
8/AVL
